# The utility of ^18^ F-FDG PET/CT for suspected recurrent breast cancer: impact and prognostic stratification

**DOI:** 10.1186/1470-7330-14-13

**Published:** 2014-04-22

**Authors:** Alexandre Cochet, Steven David, Kate Moodie, Elizabeth Drummond, Gaelle Dutu, Michael MacManus, Boon Chua, Rodney J Hicks

**Affiliations:** 1 Centre for Cancer Imaging, Peter MacCallum Cancer Centre, East Melbourne, Australia; 2 Department of Radiation Oncology, Peter MacCallum Cancer Centre, East Melbourne, Australia; 3 Centre for Biostatistics and Clinical Trials, Peter MacCallum Cancer Centre, East Melbourne, Australia; 4 The Sir Peter MacCallum Department of Oncology, the University of Melbourne, Parkville, Australia; 5 Department of Nuclear Medicine, Centre Georges-François Leclerc, 1 rue Professeur Marion, Dijon Cedex 21079, France

**Keywords:** Breast cancer, ^18^ F-FDG PET/CT, Restaging, Prognosis

## Abstract

**Background:**

The incremental value of ^18^FDG PET/CT in patients with breast cancer (BC) compared to conventional imaging (CI) in clinical practice is unclear. The aim of this study was to evaluate the management impact and prognostic value of ^18^ F-FDG PET/CT in this setting.

**Methods:**

Sixty-three patients who were referred to our institution for suspicion of BC relapse were retrospectively enrolled. All patients had been evaluated with CI and underwent PET/CT. At a median follow-up of 61 months, serial clinical, imaging and pathologic results were obtained to validate diagnostic findings. Overall Survival (OS) was estimated using Kaplan Meier methods and analyzed using the Cox proportional hazards regression models.

**Results:**

Forty-two patients had a confirmed relapse with 37 (88%) positive on CI and 40 (95%) positive on PET/CT. When compared with CI, PET/CT had a higher negative predictive value (86% versus 54%) and positive predictive value (95% versus 70%). The management impact of PET/CT was high (change of treatment modality or intent) in 30 patients (48%) and medium (change in radiation treatment volume or dose fractionation) in 6 patients (9%). Thirty-nine patients (62%) died during follow-up. The PET/CT result was a highly significant predictor of OS (Hazard Ratio [95% Confidence Interval] =4.7 [2.0-10.9] for PET positive versus PET negative for a systemic recurrence; p = 0.0003). In a Cox multivariate analysis including other prognosis factors, PET/CT findings predicted survival (p = 0.005). In contrast, restaging by CI was not significant predictor of survival.

**Conclusion:**

Our study support the value of ^18^ F-FDG PET/CT in providing incremental information that influence patient management and refine prognostic stratification in the setting of suspected recurrent breast cancer.

## Background

Breast cancer (BC) is the most commonly diagnosed cancer in women, and is the leading cause of death by cancer for women in the western world. Depending on the initial extent of the disease, approximately 30% of patients diagnosed with BC are at risk of developing loco-regional recurrence or secondary tumor dissemination to distant organs [1]. Moreover, the survival of patients who develop an isolated loco-regional recurrence differs from patients who have distant relapse. As a consequence, determination of both the locations and extent of the recurrent disease is essential to guide therapeutic decisions and estimate prognosis.

Traditionally, routine evaluation of suspected recurrent BC involves physical examination and a multi-modality Conventional Imaging (CI) approach which may include mammography, CT, MRI, and bone scintigraphy. However, this CI approach is often time-intensive and potential false-negative findings may delay appropriate therapy. Positron Emission Tomography/Computed Tomography (PET/CT) with ^18^ F-Fluorodeoxyglucose (^18^ F-FDG) is also often used in this indication, given that ^18^ F-FDG has affinity for both primary and secondary breast tumors, depending on size and aggressiveness [2-4]. Several authors have suggested that ^18^ F-FDG PET and PET/CT are more sensitive than CI for detection of recurrent BC [5-15] and can have a significant impact on the therapeutic management [5,7-9,12,16]. However, information concerning the utility of ^18^ F-FDG PET/CT for long-term prognostic stratification, when compared with CI, is limited.

Thus, the objectives of our study were to: [1] assess the incremental diagnostic performance and the impact on therapeutic management of ^18^ F-FDG PET/CT in a group of patients with a history of BC who had already been restaged by CI for identification of suspected disease relapse; [2] compare the long-term prognostic stratification of CI alone and ^18^ F-FDG PET/CT.

## Methods

### Patients

A retrospective analysis was performed on consecutive patients with a history of BC and suspicion of recurrence who were referred for ^18^ F-FDG PET/CT at our institution from January 2002 to September 2008. BC was not a funded indication of ^18^ F-FDG PET/CT during this period in Australia; therefore, clinicians usually referred patients with high suspicion of recurrence for PET/CT.

The inclusion criteria of the study were as follows: (a) a history of confirmed histologic diagnosis of primary BC treated as per local protocol; (b) CI performed no longer than 4 months prior to PET/CT and where the CI included at least a CT scan of the area of interest; (c) availability of follow-up data for a minimum of 6 months following PET/CT; (d) unequivocal determination of clinical status at the time of the last clinical follow-up.

Sixty-three patients (62 women and one man; mean age = 57 years; range = 29-86 years) were included. The median time interval from initial diagnosis to ^18^ F-FDG PET/CT was 39 months (range 5-431 months). The median time interval between CT and PET/CT was 25 days (first-third quartile: 1-52 days). Indications for PET/CT were: equivocal or suspicious CI findings (n = 28); clinical suspicion of recurrence (n = 21); restaging after completion of therapy (n = 5); routine surveillance (n = 5); and increasing levels of tumor markers (n = 4). All patients provided permission to review medical records at the time of PET/CT imaging according to our institution’s investigational review board guidelines for informed consent (protocol number 09/78).

### PET/CT acquisition and processing

Whole-body PET was acquired sequentially using a dedicated PET/CT system (Discovery LS PET/ 4-slice helical CT or Discovery STE/ 8-slice helical CT, General Electric Medical Systems, Milwaukee, WI) combining a multidetector CT scanner with a dedicated, full-ring PET scanner with bismuth germanate crystals. Patients were instructed to fast except for glucose-free oral hydration for at least 6 hours before injection of 300-400 MBq of ^18^ F-FDG. PET was performed 60 min following ^18^ F-FDG injection. Blood glucose levels were measured before the injection of the tracer to ensure levels below 10 mmol/l. Transmission data used for attenuation correction were obtained from a low-dose non diagnostic CT acquisition (140 kVp and 40-120 mA), without contrast enhancement. Attenuation corrected PET images were reconstructed with an iterative reconstruction (ordered-subset expectation maximization algorithm). Orthogonal CT, PET, and fused PET/CT images were displayed simultaneously on a GE Xeleris Workstation. The PET data were also displayed in a rotating maximum-intensity projection.

An experienced nuclear medicine physician generated a clinical report after reviewing PET images, low-dose CT images, fused PET/CT images, previous imaging results and clinical information. Standard uptake values were not routinely measured. Once issued, the PET/CT report was not reinterpreted in the light of subsequent clinical information.

### Image interpretation and classification

A total of 188 clinical, imaging and pathological procedures were performed (3 ± 1.4 per patients), including chest CT (n = 59), abdominopelvic CT (n = 44), whole-body bone scan (n = 30), clinical examination (n = 17), pathology (n = 9), abdominal ultrasound (US) (n = 7), MRI (n = 7), mammogram or breast US (n = 6), chest radiography (n = 5), other (n = 5).

Written clinical reports of conventional images and PET/CT were reviewed and classified as (a) negative if imaging tests were negative for disease; (b) equivocal, when abnormal findings were present on any imaging test but were not interpreted as suspicious for malignancy; (c) positive, if any result was clearly described as suspicious or consistent with malignancy. Negative and equivocal findings were combined as negative for the analysis.

In cases where recurrence was reported on CI or PET/CT, the location of relapse was also determined, and classified as loco-regional (ipsilateral breast, ipsilateral axillary, internal mammary or supraclavicular node station) or systemic (contralateral node station or distant metastasis). The final diagnosis of disease recurrence and location of disease was confirmed by histologic examination in 13 patients (21%). For the remaining patients, evidence of progression within 6 months of clinical and/or imaging follow-up was considered to indicate a site of disease relapse, whereas no evidence of progression after at least 6 months of follow-up was considered to confirm absence of active disease at that site.

### Assessment of impact

Referring physicians were asked to record a pre-PET/CT management plan before PET/CT results on our routine clinical request form. The actual post-PET/CT management plan and treatment intent were determined from the medical record or by contacting the referring clinician.

The impact of PET/CT on management was considered “high” when the treatment intent or modality was changed (e.g. from palliative to curative treatment or from surgery to radiotherapy) [17]. The impact was considered as “medium” when the method of treatment delivery was changed (e.g. radiation treatment volume and/or dose fractionation) [17]. When the PET/CT results did not indicate a need for change, the impact was considered to be “low”. PET/CT was considered to have had “no impact” when the management chosen conflicted with post-PET/CT disease extent on the basis of a synthesis of all available information.

### Follow-up

After PET/CT, progress updates were obtained from the medical record, family physician, or treating oncologist. When relevant, details of the date and cause of death were obtained. The disease status at the time of death was recorded.

### Statistical analysis

Estimates of OS at 2 and 5 years were computed using the Kaplan Meier method, a log-rank test was used to analyze the effect of CI and PET/CT results on OS. Two Cox regression analyses were performed to assess the impact of PET/CT and CI on OS controlling for clinical variables and using a backward elimination process. Triple negative status of the primary tumor was not included in the multivariate model because this information was available for only 38 patients. For percentages such as PPV, NPV, sensitivity and specificity, a Blyth-Still-Casella 95% confidence interval (CI) was calculated. Diagnosis performance results were compared using McNemar tests, Fisher exact tests and a likelihood ratio, which summarizes how many times more likely patients with the disease are to have that particular result than patients without the disease. According to the fact that patients were selected on the basis of CI, many patients with unequivocal systemic relapse on CI would likely not have been referred for PET/CT. Given the likely pre-test selection bias, positive and negative predictive values were considered as more relevant comparators of diagnostic performance when compared with sensitivity and specificity.

## Results

Patient characteristics at the time of initial diagnosis are summarized in Table 1.

**Table 1:** Patient characteristics at the time of initial diagnosis

**Characteristics**	**Number of patients (%)**
	**(sample size = 63)**
Localisation	
Right	30 (48)
Left	29 (46)
Bilateral	4 (6)
Histology	
Ductal	42 (67)
Lobular	11 (17)
Other	7 (12)
Unknown	3 (5)
Histological grade	
1 and 2	28 (44)
3	25 (40)
Unknown	10 (16)
Lymphovascular invasion	
+	22 (35)
-	23 (36)
Unknown	18 (29)
Estrogen receptor status	
+	34 (54)
-	20 (32)
Unknown	9 (14)
Progesterone receptor status	
+	25 (40)
-	28 (44)
Unknown	10 (16)
HER2 status	
+	9 (14)
-	29 (46)
Unknown	25 (40)
T stage	
1	29 (46)
2	17 (27)
3	10 (16)
4	3 (5)
X	4 (6)
N status	
+	29 (46)
-	31 (49)
X	3 (5)

HER-2 = Human Epidermal Growth Factor Receptor-2.

### Diagnostic performance

Relapse involving at least one site was confirmed in 42 of the 63 patients (67%). CI was positive for disease in 37 of these patients, yielding a patient sensitivity of 88%, whereas PET/CT was positive for disease in 40, corresponding to a patient sensitivity of 95%.

Table 2 shows comparison of extent of suspected relapse as assessed before and after PET/CT. Downstaging by PET/CT was confirmed to be correct in 12/14 patients (one patient had a suspicious bony lesion on bone scan that was non ^18^ F-FDG-avid, but confirmed to be malignant; one patient showed suspicious mediastinal lymph nodes on CT that were non ^18^ F-FDG-avid but confirmed to be metastasis of breast cancer by pathology), while upstaging with PET/CT was confirmed in 5/5 patients.

**Table 2:** Comparison of extent of suspected relapse as assessed before and after PET/CT

**Pre-PET/CT extent of relapse**	**Post-PET/CT extent of relapse**				**% less disease by PET/CT**	**% same status**	**% more disease by PET/CT**
	**Negative**	**LR only**	**Systemic**	**Total**			
Negative	8	1	3	12	-	66	33
LR only	1	6	1	8	12.5	75	12.5
Systemic	12	1	30	43	30	70	-
Total	21	8	34	63	22	70	8

LR = Loco-Regional; PET/CT = Positron Emission Tomography/Computerized Tomography.

On final diagnosis, 20 patients (32%) had a loco-regional recurrence and 36 (57%) had a systemic recurrence (14 patients had both loco-regional and systemic recurrence). CI was truly positive for loco-regional recurrence in 8/20 patients (40%) and systemic recurrence in 30/36 patients (83%). PET/CT was truly positive for loco-regional recurrence in 20/20 patients (100%) and systemic disease in 32/36 patients (89%). Among the 20 patients experiencing loco-regional recurrence, only 3 had local relapse only, for whom both PET/CT and CI were positive. PET/CT detected regional relapse non-detected by CI in 12 patients (3 in axillary nodes only, 9 in extra-axillary nodes). PET/CT had significantly higher positive predictive values when compared with CI for determination of loco-regional, systemic and global recurrence, and higher negative predictive value for loco-regional and global recurrence (Table 3). ^18^ F-FDG PET/CT found incidental malignancies in 2 patients (one patient had a primary esophageal cancer and one patient had a gastrointestinal stromal tumor).

**Table 3:** Diagnostic accuracy of conventional imaging (CI) and PET/CT in detecting relapse of disease in all patients, according to the histological type, and for locoregional and systemic recurrence

		**Negative predictive value**			**Positive predictive value**			**Likelihood**
	**n**		**95% CI**	**p**		**95% CI**	**p**	Ratio*
*All patients:*	63							
Conventional Imaging		7/13 (**54%**)	29–82		35/50 (**70%**)	59–84		1.3
PET/CT		19/22 (**86%**)	70–98	**0.05**	39/41 (**95%**)	84–99	**0.003**	9.8
*Locoregional recurrence:*	63							
Conventional Imaging		41/53 (**77%**)	65–88		8/10 (**80%**)	44–96		8.6
PET/CT		42/42 (**100%**)	93–100	**<0.001**	20/21 (**95%**)	78–100	**0.24**	43
*Systemic recurrence:*	63							
Conventional imaging		14/19 (**74%**)	50–89		31/44 (**70%**)	56–82		1.8
PET/CT		26/29 (**90%**)	74–97	**0.24**	33/34 (**97%**)	85–100	**0.002**	24.8

CI = Confidence Interval; PET/CT = Positron Emission Tomography/Computerized Tomography. Significant p values in bold.

*likelihood ratio = sensitivity/ (1-specificity).

### Impact on management

The PET/CT results had a high impact on management in 30 patients (48%) of whom the treatment intent was modified in 24 patients (including from invasive diagnosis to observation for 11 patients, according to negative PE/CT results despite suspicious CI findings). For the 6 remaining patients, the treatment intent was not modified after PET/CT (palliative for 5 patients, curative for 1 patient), but the modalities of therapy were changed.

PET/CT had a medium impact on management in 6/63 patients (9%). Management changes in these patients primarily included changes in radiation treatment volume as a result of more extensive disease detected by PET/CT. All these patients had a palliative treatment intent, which was not modified by PET/CT.

PET/CT had a low impact (i.e. did not change the planned management) in 27/63 patients (43%) for whom the relapse extent was concordant with that found on CI (19 patients) or the documentation of a different distribution of disease did not alter the planned treatment (2 patients), or both PET/CT and CI findings were negative (6 patients). In no case was the PET/CT result apparently ignored.

### Prediction of survival by CI and PET/CT

Survival data were analyzed with a close-out date of September, 4th 2010. The median follow-up time was 5.1 years (range 0.8-8.6 years). All 63 patients entered into the study had a known status at the close-out date. All patients with negative PET/CT findings were followed for a minimum of 2 years after the scan (except one patient who died 2 months after the scan, because of complications of an esophageal primary tumor discovered on PET/CT).

**Figure 1:**
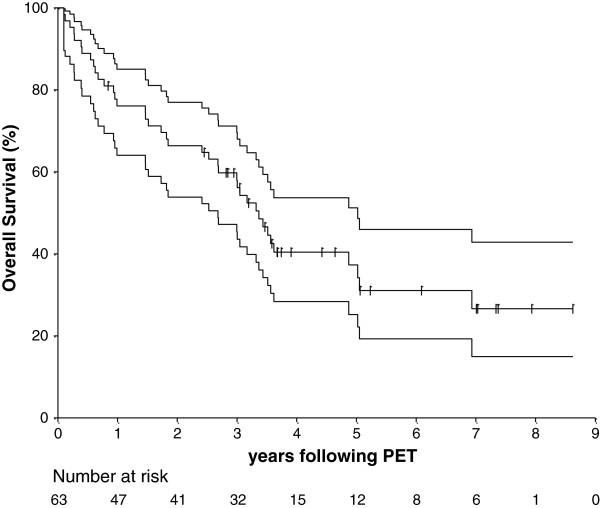
Estimated overall survival (+/- 95% confidence interval) for all 63 patients.

Thirty-eight patients (60%) were deceased with a median survival of 3.4 years (95% CI 2.5 to 5.0 years) (Figure 1). The estimated 2-year OS was 66.4% (95% CI 53.2% to 76.6%) and the estimated 5-year OS was 37.3% (95% CI 24.3% to 50.3%).

**Figure 2:**
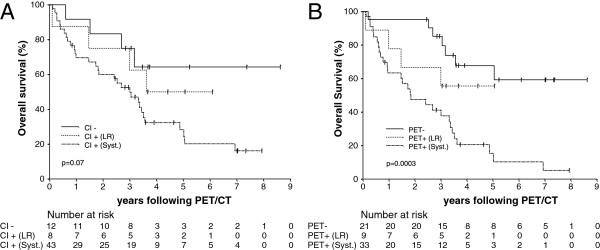
**Overall survival by staging technique. (A)** Kaplan-Meier estimate of overall survival (OS) stratified by pre-PET/CT (Conventional imaging) status. **(B)** Kaplan-Meier estimate of OS stratified by post-PET/CT status. CI = Conventional Imaging; LR = loco-regional recurrence only; Syst = systemic recurrence.

**Figure 3:**
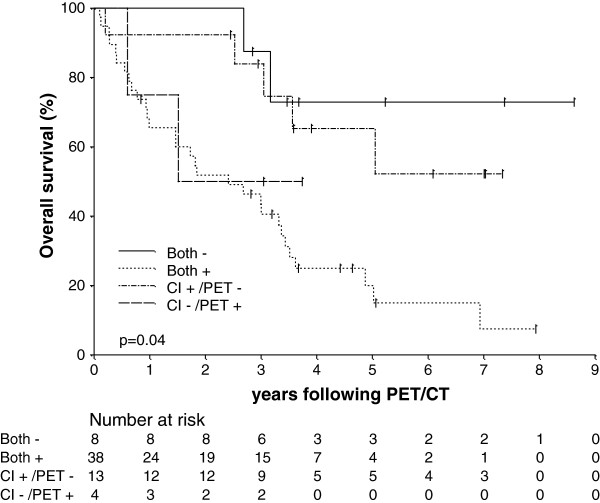
Kaplan-Meier estimate of overall survival (OS) stratified by combination of Conventional imaging (CI) and PET/CT results.

On univariate analysis, PET/CT status (negative, positive LR or positive systemic) was strongly associated with survival (log-rank test: p = 0.0003 for the entire model; p = 0.0001 for comparison of negative results and positive for systemic disease; p > 0.05 for other single comparisons) (Figure 2). Patients with systemic disease according to PET/CT had a 4.7-fold in the risk of death when compared with patients with negative PET/CT findings, while patients with only loco-regional recurrence had a 2-fold increase in the risk of death (Table 4). In contrast, CI status (negative, positive LR or positive systemic) did not significantly predict OS (log-rank test: p = 0.07 for the entire model; p > 0.05 for all single comparisons) (Figure 2). Moreover, patients with positive CI findings but negative PET/CT findings had similar estimated survival to patients in whom both tests were negative, whereas patients with negative CI findings but positive PET/CT results had comparable estimated survival to patients in whom both procedures were positive for recurrence (Figure 3). 

**Table 4:** Univariate predictors of overall survival

		**Cox regression**		
		**Relative hazard rate**		
	**No. pts**	**HR**	**95% CI**	**Logrank test**
All patients	63			
Time between diagnosis and PET/CT				0.94
0 to 3 years	33	1.00	(Reference)	
4 to 9 years	16	1.13	(0.54 – 2.37)	
10+ years	14	1.07	(0.47 – 2.46)	
Histological type				**0.018**
Invasive ductal carcinoma	42	1.00	(Reference)	
Invasive lobular carcinoma	11	2.83	(1.34 – 6.02)	
Mixed, Mucinous and other	7	1.66	(0.66 – 4.40)	
T				**0.0004**
T1	29	1.00	(Reference)	
T2	17	2.94	(1.33 – 6.50)	
T3/T4	13	4.70	(1.99 – 11.05)	
N				0.27
N	31	1.00	(Reference)	
N+	29	1.43	(0.75 – 2.75)	
Stage at diagnosis				**0.009**
Stage I	19	1.00	(Reference)	
Stage II	19	3.05	(1.17 – 7.90)	
Stage III/IV	21	3.68	(1.51 – 9.00)	
Histological grade				0.92
1 and 2	28	1.00	(Reference)	
3	25	1.04	(0.52 – 2.05)	
ER status				0.067
Positive	34	1.00	(Reference)	
Negative	20	1.87	(0.95 – 3.68)	
PR status				0.29
Positive	25	1.00	(Reference)	
Negative	28	1.45	(0.73 – 2.88)	
HER-2 status*				0.85
Positive	9	1.00	(Reference)	
Negative	29	1.09	(0.44 – 2.73)	
Triple negative status*				**0.0003**
At least one Positive	27	1.00	(Reference)	
Triple Negative	9	5.28	(1.99 – 14.00)	
CI findings				0.07
Negative	12	1.00	(Reference)	
Positive - loco regional	8	1.54	(0.38 – 6.16)	
Positive - systemic	43	2.89	(1.02 – 8.18)	
PET/CT findings				**0.0003**
Negative	21	1.00	(Reference)	
Positive - loco regional	9	2.02	(0.59 – 6.98)	
Positive - systemic	33	4.71	(2.03 – 10.93)	

*The information is available for only 38 patients.

CI = Confidence Interval; ER = Estrogen Receptor; HER-2 = Human Epidermal Growth Factor Receptor-2; HR = Hazard Ratio; PET/CT = Positron Emission Tomography/Computerized Tomography; PR = Progesterone Receptor. Significant p values in bold.

Initial stage of the disease at the time of diagnosis, histological type of cancer and triple negative status (hormone receptor and HER-2 negativity) were also predictors of survival on the univariate analysis (Table 4). Time between initial diagnosis and the restaging PET/CT, initial histological grade, estrogen receptor status, progesterone receptor status and overexpression of Human Epidermal Growth Factor Receptor-2 (HER-2) were not significant predictors of survival. On multivariate analysis, positive PET/CT findings (for loco-regional and/or systemic recurrence) remained an independent predictor of OS when adjusting for age, histological subtype and initial stage (model 1, Table 5). In contrast, positive CI findings did not independently predict OS (model 2, Table 5).

**Table 5:** Multivariate predictors of overall survival

	**No of observed deaths**	**HR**	**95% CI**	**P**
**Model 1**	36/58			
Age (per 10 years)		1.4	1.1–1.7	**0.02**
Invasive ductal carcinoma vs. other type		2.6	1.3–5.3	**0.007**
Initial stage II, III, IV vs. I		4.1	1.7–10.3	**0.002**
PET/CT positive vs negative		3.5	1.5–8.2	**0.005**
**Model 2**	36/58			
Age (per 10 years)		1.4	1.1–1.8	**0.01**
Invasive ductal carcinoma vs. other		2.9	1.4–5.8	**0.003**
Initial stage II, III, IV vs. I		5.4	2.1–13.4	**<0.001**
Conventional Imaging positive vs. negative		2.0	0.7–5.7	**0.22**

CI = Confidence Interval; HR = Hazard Ratio; PET/CT = Positron Emission Tomography/Computerized Tomography. Significant p values in bold.

## Discussion

For patients with possible recurrent breast cancer (BC), early detection and adequate localization of recurrent disease are essential for guiding optimal therapy and prognostication. Patients with isolated loco-regional recurrence are able to benefit from curative salvage therapy, whereas palliative treatment is generally indicated for patients with distant relapse.

Several studies have shown the relevance of ^18^ F-FDG PET/CT in detecting distant metastasis in patients with clinical suspicion of recurrence [6,8,12,13,18-20], and in patients with documented loco-regional recurrence [5]. Our study confirmed that ^18^ F-FDG PET/CT is an accurate technique for the appropriate detection of relapse, when compared with CI alone. Of particular economic and clinical importance was the observation that 12 patients (19%) in this series who were suspected to have relapsed by conventional evaluation subsequently received no active treatment after a negative ^18^ F-FDG PET/CT evaluation and demonstrated an excellent prognosis. In contrast, of the 8 patients with negative CI findings, 1 patient was found to have locoregional relapse and 3 patients were found to have systemic recurrence on ^18^ F-FDG PET/CT. In the current study, ^18^ F-FDG PET/CT had an impact on therapeutic management in 57% of patients; in particular, the treatment intent was changed in 38% of patients. This result is consistent with other studies which showed the important impact of ^18^ F-FDG PET/CT on therapeutic management in patients with suspicion of recurrent BC [5,8,12,18,19]. Most of these previous studies reported that ^18^ F-FDG PET/CT was highly accurate for detecting recurrent disease in patients with negative or inconclusive CI findings [5,18,19]. In contrast, in our study, CI findings were consistent with relapse in the majority of patients (51/63) but ^18^ F-FDG PET/CT downstaged 12 of them, providing a better negative predictive value when compared to CI alone (Table 3). These findings suggested that ^18^ F-FDG PET/CT was not only effective for early detection of relapse in patients with negative CI findings, but also yielded a better characterization of CI findings in patients with a high suspicion of relapse.

As BC was not a funded indication of ^18^ F-FDG PET/CT in Australia during the study period, referring clinicians were likely to use PET/CT in patients for whom there was clinical uncertainty with respect to the appropriate management. Although this patient selection could have introduced biases in the evaluation of the impact of PET/CT findings on patient management, it also showed the value of ^18^ F-FDG PET/CT in patients whose disease status could not be adequately determined using CI alone.

The more accurate prognostic information derived from PET/CT results when compared with CI findings underpinned the value of PET/CT in the management of patients with recurrent BC. Both ^18^F-FDG PET/CT and CT are not optimal modalities for detection of local recurrence, when compared with mammography, ultrasound or MRI. However, detection of additional distant metastases in patients with documented loco-regional recurrence is essential in order to optimize management and stratify prognosis. Of note was the favourable survival of patients with isolated loco-regional recurrence according to PET/CT, when compared with patients with systemic relapse.

In our study, CT performed with PET was not of diagnostic quality. However, all patients included in this study had diagnostic CT before undergoing FDG PET/CT.

While Dirisamer et al. showed in a retrospective study that the association of FDG PET and contrast-enhanced (ce) CT could improve restaging of breast cancer when compared with ceCT or FDG PET alone [6], there is no evidence in the literature of the superiority of FDG PET/ceCT for restaging of breast cancer when compared with FDG PET associated to non-diagnostic, low dose CT. In our study, the outcome data validate our approach in that FDG PET/CT results were more often correct than conventional imaging (including diagnostic CT) when discordant, and stratified prognosis whereas conventional imaging did not (Figure 2). If we hadn’t ignored the conventional imaging findings (including diagnostic CT) when discordant with FDG PET/CT, the accuracy wouldn’t have been enhanced and the prognostic value of FDG PET/CT incorporating a low dose, non-contrast CT wouldn’t have been superior to that of conventional imaging. These results have implications for the reporting of FDG PET combined with ceCT, which is performed by some facilities as a routine procedure and suggest that significant clinical weight should be placed on the PET findings even when discordant with the ceCT appearances.

One of the limitations of our study was that the CI procedures were not standardized and were selected on the basis of clinical findings. However, this represented routine clinical practice and did not detract from the results. Although comparison of a masked reading of PET/CT with a masked reading of CI techniques might be appropriate if PET/CT were to be suggested as a replacement for CI, the main purpose of this study was to evaluate the incremental diagnostic and prognostic value of PET/CT in routine practice. The results of this retrospective study would, however, justify a randomized trial in which patients with clinical risk or suspicion of relapse would be stratified to have either CI or FDG PET/CT as the initial restaging procedure [21]. Finally, we did not compare PET/CT and CI findings with histopathological findings in most patients. For 50 patients (79%), the final disease status was determined clinically and/or with follow-up imaging. Nevertheless, since most of the patients were followed up for a long period of time, the survival analysis would be the best validation of diagnostic accuracy.

## Conclusion

Our findings support the value of ^18^ F-FDG PET/CT in providing incremental information that influence patient management and refine prognostic stratification in the setting of suspected recurrent breast cancer. The prognostic stratification provided by this technique emphasizes the crucial role of ^18^ F-FDG PET/CT in optimizing treatment choices in this setting. Further multicentric studies are needed to confirm this role in particular in patients with high suspicion of relapse.

## Competing interests

The authors declare that they have no competing interests.

## Authors’ contributions

AC, SD, KM and RJH were involved in the concept and design of the study. AC SD, and ED were involved in data collection, history review and follow-up. KM was the imaging lead on the study and with RJH verified classification of scan results. GT was the study statistician and contributed to analysis of the primary data. SD, MM and BC were the clinical leads for assessing the impact criteria and verifying classifications of PET/CT impact. All authors read and approved the final manuscript.
